# Anti-tuberculosis activity of oleanolic and ursolic acid isolated from the dichloromethane extract of leaves from *Duroia macrophylla*

**DOI:** 10.1186/1753-6561-8-S4-P3

**Published:** 2014-10-01

**Authors:** Daiane Martins, Lillian Lucas Carrion, Daniela Fernandes Ramos, Kahlil Schwanka Salomé, Pedro Eduardo Almeida da Silva, Barison Andersson, Cleverson Agner Ramos, Cecilia Veronica Nunez

**Affiliations:** 1 Bioprospection and Biotechnology Laboratory, National Research Institute of Amazonia - INPA, Manaus, AM, Brazil; 2 Mycobacterial Laboratory, Federal University Foundation of Rio Grande - FURG, Rio Grande, RS, Brazil; 3 NMR Laboratory, Department of Chemistry, Federal University of Paraná - UFPR, Curitiba, PR, Brazil; 4 Departament of Morphology, Federal University of Amazonas, Manaus, Brazil; 5 Bioprospection and Biotechnology Laboratory, National Research Institute of Amazonia - INPA, Manaus, AM, Brazil

## Introduction

Tuberculosis a major cause of death worldwide, is an infectious disease caused by Mycobacterium tuberculosis. An increase in drug-resistant tuberculosis cases and the emergence of additional resistant strains and coinfections with HIV has stimulated the search for and development of new anti-TB drugs [1]. The wide variety of natural products chemical structures plays a major role on the development of new antimycobacterial drugs generations. *Duroia macrophylla *is an endemic plant of the Amazon Forest [2]. To the best of our knowledge, no chemical or biological investigations other than ours [3,4] have been carried out on this species as of yet. Hence this work aims to evaluate the antimycobacterial activity of their extracts and isolate and identify the substances present in *D. macrophylla *active extracts.

## Methodology

Its leaves and branches were collected twice and extracted with dichloromethane and methanol. All extracts were subjected to phytochemical investigation and terpenes and flavonoids were found in all dichloromethane and methanol extracts, respectively.

## Results and discussion

Methanol extracts from both branches (1^st ^collection) and leaves (2^nd ^collection) presented hydrolyzed tannins, yet alkaloids were only detected in the dichloromethane and methanol extracts from branches at the 2^nd ^collection. Phenol compounds were found in both dichloromethane extracts collections. The action of every extract was assayed against *Mycobacterium tuberculosis *(RMPr, H37Rv and INHr strains), showing the dichloromethane extract from leaves (1^st ^collection) the major biological activity, with a MIC of 6.25 μg/mL for INHr strain, 25.0 μg/mL for RMPr strain and ≤ 6.25 μg/mL for H37Rv strain. The chromatographic fractioning of dichloromethane extract from leaves (1^st ^collection) yielded the isolation of two triterpenes: oleanolic and ursolic acids, which were identified by NMR analysis and reported for the first time in *Duroia *genus. The highest activity of the dichloromethane extract from leaves (1st collection) in this work could be attributed to the presence of terpenes. Several studies, showed terpenes to be responsible for the antimycobacterial activity. The high lipophilicity of terpenes is probably the main factor that allows their penetration through the mycobacterial cell wall. Literature data reported that oleanolic acid has a synergistic effect when combined with isoniazid, rifampicin or ethambutol (first line antitubercular drugs) [5].

## Conclusion

Hence, more thorough studies are necessary to find what substances should be mixed in order to attain the desirable antimycobacterial activity.
